# NLR family pyrin domain containing 3 (NLRP3) inflammasome gene polymorphism rs7512998 (C>T) predicts aging-related increase of blood pressure, the TAMRISK study

**DOI:** 10.1186/s12979-015-0047-7

**Published:** 2015-10-31

**Authors:** Tarja Kunnas, Kirsi Määttä, Seppo T. Nikkari

**Affiliations:** Department of Medical Biochemistry, University of Tampere Medical School and Fimlab laboratories, Tampere, Finland

**Keywords:** NLRP3, Human, Hypertension, Single nucleotide polymorphism, Health surveys

## Abstract

**Background:**

The activation of NLR family pyrin domain containing 3 (NLRP3) inflammasome by cellular stress leads to activation of the inflammasome, and NLRP3 gene polymorphisms have been associated with autoinflammatory diseases. Inflammasomes have also been implicated in the initiation or progression of metabolic disorders such as atherosclerosis, type 2 diabetes and obesity. The association of NLRP3 genetic variant rs7512998 with blood pressure and hypertension was studied in a 50-year-old Finnish cohort with a subpopulation who had available data on blood pressure measurements also at the age of 45 years.

**Results:**

NLRP3 gene polymorphism rs7512998 C-allele was associated with higher systolic (*p* = 0.006) and diastolic (*p* = 0.011) blood pressure compared to the TT-genotype carriers in 50-year-old subjects. In addition, by analysis of variance for repeated measures between ages of 45- and 50 years there was a significant time by genotype interaction; blood pressure increased more in subjects with the C-allele both in systolic (*p* = 0.035) and diastolic (*p* = 0.012) values. However, no association with diagnosed hypertension was found.

**Conclusion:**

We report for the first time that NLRP3 gene polymorphism rs7512998 was associated with systolic and diastolic blood pressure in 50-year-old subjects. In addition, an effect of this variation upon blood pressure was seen in these same subjects in a 5-year follow-up from a 45-year-old cohort to 50 years of age.

## Background

There is increasing evidence that inflammasomes are involved in the initiation or progression of diseases including metabolic disorders such as atherosclerosis, type 2 diabetes and obesity [1]. Inflammasomes are large multimeric protein complexes and most of them are formed with one or two family members of the nucleotide-binding, leucine-rich repeat containing proteins (NLR). Of the NLR family, pyrin domain containing (NLRP) subfamily member NLRP3 is the most studied and appears as the major sensor for intracellular danger signals [2–5]. The activation of NLRP3 by cellular stress leads to inflammasome activation catalyzing the formation of active proinflammatory cytokines, interleukin (IL)-1β and IL-18, which are then secreted [6–8]. Previous studies have suggested an association of NLRP3 gene polymorphisms and autoinflammatory diseases, such as type 1 diabetes and inflammatory bowel disease [4, 5]. Genetic variability within NLRP3 may be also important in the pathophysiology of abdominal aortic aneurysms [9]. Multiple mutations in the NLRP3 gene are associated with markedly elevated serum IL-1β levels [10, 11]. A pivotal role of the inflammasome has been suggested also in gout, where monosodium urate crystals activate NLRP3 [12]. In fact, genetic frequency of NLRP3 intronic variant rs7512998 was significantly different between gout and control patients [13]. There have been suggestions that hypertension could have also an inflammatory background [14]. The SNP rs4986790 of the toll-like receptor (TLR) four, which plays a key role in the innate immune response and also participates in NLRP3 priming, has recently been shown to associate with age-dependent blood pressure increase in patients with coronary artery disease [15]. However, no previous studies have investigated the association of NLRP3 gene polymorphisms with elevated blood pressure. Therefore, we wanted to assess the role of NLRP3 gene rs7512998 variants in a Finnish 50-year-old population, by analyzing cohorts from the Tampere adult population cardiovascular risk study (TAMRISK) [16].

## Results

Clinical characteristics of the cases (325) and controls (444) at the age of 50 years are presented in Table 1. The case group of hypertensive subjects was compared to healthy controls. Cases had higher body mass index (BMI), fasting glucose, and systolic and diastolic blood pressure compared to controls. In the whole study population the frequencies of the NLRP3 gene rs7512998 genotypes were 0.02 for CC (*n* = 16), 0.28 for TC (*n* = 216) and 0.70 (*n* = 537) for TT. These frequencies were in Hardy-Weinberg equilibrium (*p* = 0.287), and did not differ between cases and controls.

**Table 1:** Clinical characteristics (means ± SD) of the study population at the age of 50 years

	Cases (*n* = 325)	Controls (*n* = 444)	*P*
Age (years)	50 ± 0	50 ± 0	
Gender (male) %	59	63	0.279
Blood pressure medication (%)	73	0	<0.000
Smoking (%)	27	25	0.588
Body mass index (kg/m2)	28.8 ± 5.1	25.5 ± 3.6	<0.000
Cholesterol (mmol/l)	5.40 ± 0.98	5.37 ± 0.88	0.789
Glucose (mmol/l)	5.17 ± 1.30	4.86 ± 0.54	<0.000
Systolic blood pressure (mm Hg)	142.7 ± 16.7	129.3 ± 14.8	<0.000
Diastolic blood pressure (mm Hg)	92.8 ± 8.9	84.4 ± 9.1	<0.000
NLRP3 gene polymorphism rs7512998 (CC/TC/TT) %	1.5/28.9/69.6	2.5/27.8/69.7	0.647
rs7512998 (CC+TC/TT) %	30.4/69.6	30.3/69.7	0.626

The statistical analysis of clinical characteristics between different genotype groups for NLRP3 gene are given in Table 2. Subjects with C allele had significantly higher systolic (*p* = 0.006) and diastolic (*p* = 0.011) blood pressure at the age of 50 years than those homozygous for the T allele. The difference between subjects with the C allele and genotype TT was 3.4 mmHg in systolic blood pressure and 1.9 mmHg in diastolic blood pressure. This difference in blood pressure was not seen at the age of 45 years. At the ages of 45 and 50 years there were no differences between the genotype groups in prevalence of hypertension, blood pressure medication, BMI, serum cholesterol or glucose levels. Multivariate analysis confirmed NLRP3 gene rs7512998 genotype as an independent predictor of both systolic (Table 3) and diastolic blood pressure (Table 4) at the age of 50 years. In the same model, male gender and BMI were also such predictors.

**Table 2:** Clinical characteristics (means ± SD) of the study population stratified according to NLRP3 gene polymorphism rs7512998. *P* values <0.05 are in bold

	CC + TC	TT	*p*
N at 50 (%)	232 (30)	537 (70)	
Hypertension (%)	41.2	42.3	0.807
Blood pressure medication (%)	28.1	32.2	0.273
Smoking (%)	23	28	0.128
Systolic blood pressure (mmHg)	137.6 (18.0)	134.0 (16.2)	0.006
Diastolic blood pressure (mmHg)	89.3 (10.2)	87.4 (9.6)	0.011
Body mass index (kg/m^2^)	27.3 (4.8)	26.8 (4.6)	0.196
Cholesterol (mmol/l)	5.4 (0.9)	5.4 (1.0)	0.959
Glucose (mmol/l)	5.0 (0.6)	5.1 (1.3)	0.309
N at 45 (%)	199 (29)	479 (71)	
Hypertension (%)	12.6	16.2	0.253
Blood pressure medication (%)	7.6	12.4	0.083
Smoking (%)	42	42	0.911
Systolic blood pressure (mmHg)	133.3 (16.1)	132.4 (13.6)	0.417
Diastolic blood pressure (mmHg)	85.3 (9.5)	85.2 (9.2)	0.906
Body mass index (kg/m^2^)	26.7 (4.1)	26.3 (4.3)	0.219
Cholesterol (mmol/l)	5.5 (1.1)	5.5 (1.1)	0.617
Glucose (mmol/l)	5.4 (0.8)	5.2 (0.9)	0.449

**Table 3:** Linear regression for systolic blood pressure at the age of 50 years

Parameter	Coefficient B	Std. error	Beta	*P*
Gender (Female = 0, Male = 1)	2.844	1.165	0.082	0.015
Body mass index	0.390	0.132	0.106	0.003
Hypertension (No = 0, Yes = 1)	12.376	1.228	0.359	<0.001
NLRP3 SNP 7512998 (TT = 0, CC+TC = 1)	3.361	1.236	0.091	0.007

**Table 4:** Linear regression for diastolic blood pressure at the age of 50 years

Parameter	Coefficient B	Std. error	Beta	*P*
Gender (Female = 0, Male = 1)	3.206	0.655	0.158	<0.001
Body mass index	0.388	0.074	0.181	<0.001
Hypertension (No = 0, Yes = 1)	7.278	0.690	0.363	<0.001
NLRP3 SNP 7512998 (TT = 0, CC+TC = 1)	1.623	0.694	0.075	0.020

When the 45- and 50 year follow-up data of blood pressure measurements in Table 2 was evaluated by analysis of variance for repeated measures, there was a significant time by genotype interaction; blood pressure increased more in subjects with the C-allele both in systolic (*p* = 0.035) and diastolic (*p* = 0.012) values.

## Discussion

We report that C-allele of the NLRP3 gene polymorphism was significantly associated with higher blood pressure compared to genotype TT at the age of 50 years. To our knowledge, there are no previous studies showing such an association. The C-allele of rs7512998 has previously been shown to be more common in gout patients compared with controls, although 16 other NLRP3 gene polymorphisms showed no such association [13]. In the present study there was no association of C-allele with blood pressure at the age of 45 years, but there was a significant time by genotype interaction; blood pressure increased more in subjects with the C-allele both in systolic and diastolic values by the age of 50 years. It is known that a single gene variation on in GWAS studies affects blood pressure +1.16 mmHg at the most [17]. At the age of 50 years, the difference between subjects with the C allele and genotype TT in systolic blood pressure was +3.4 mmHg in systolic- and +1.9 mmHg in diastolic blood pressure. The effect was not masked by blood pressure medication, although practically all of the subjects who had diagnosed hypertension were on medication at this age. It is to be noted that the NLRP3 gene polymorphism was an independent predictor of both systolic and diastolic blood pressure independently of prior diagnosed hypertension, sex and BMI by multivariate analysis.

The observed increase in blood pressure in subjects with the NLRP3 gene rs7512998 C-allele with aging may be linked to modified activation of NLRP3 by aging-related reactive oxygen species (ROS) production by damaged mitochondria. This process of inflammasome activation through NLRP3 is present in many age‐related conditions, such as obesity, and type 2 diabetes [18]. An anti-oxidant has been shown to ameliorate experimental pulmonary artery hypertension via inhibiting NLRP3 inflammasome signal pathway in rats [19]. NLRP3 causes activation of inflammatory caspases which activate precursors of IL‐1β and IL‐18 and stimulate their secretion. Among other cytokines, IL-1β and IL‐18 are known to be an important modulators in artery wall inflammation and acceleration of atherosclerosis [20–22]. A cholesterol crystal-induced further NLRP3 inflammasome activation in macrophages may represent an important link between cholesterol metabolism and inflammation in atherosclerotic lesions [23]. The NLRP3 gene polymorphism Q705K (rs35829419) has been shown to confer a protective effect against the risk of developing MI in females [24].

The toll-like receptors (TLR) are transmembrane proteins that are important for immune surveillance at the cell surface and endolysosomal compartments, recognizing a wide range of microbial structures and endogenous danger signals. In most cell types inflammasome activation commonly includes priming with a TLR agonist, such as lipopolysaccharide (LPS), which induces elevated expression of NLRP3 [25]. Once primed, NLRP3 may respond to danger signals and form the NLRP3 inflammasome. Our present results and a recent similar observation of an association with age-dependent blood pressure increase with TLR4 SNP rs4986790 genotype [15] may suggest that increase of inflammasome action is related to increase in blood pressure.

There was a gender bias since in multivariate analysis male gender was a predictor of both systolic and diastolic blood pressure. However, multivariate analysis also showed that C-allele of the NLRP3 rs7512998 gene polymorphism was significantly associated with higher blood pressure, independently of gender and BMI. Life-style factors such as smoking and exercise contribute to inflammation, metabolic syndrome and blood pressure increase. However, in regard to smoking and BMI, there were no differences between genotype groups.

The study group was restricted to residents of a large city in Finland, posing a challenge to how broadly one can apply the findings. Since the study subjects are from a restricted genetic pool (Finnish Caucasian), the findings might not be extrapolated to different genetic populations. Replication studies should be called for to explore more fully the findings with further consideration of the molecular mechanisms. In addition, each registration of blood pressure was made at one examination visit only, and most of the subjects with hypertension were already on medication by the age of 50 years. It is also clear that information on medication had not been provided by some of the participants with hypertension, since the percentage of these with recorded blood pressure treatment was only 73 %.

## Conclusions

In conclusion, our results indicate that NLRP3 gene polymorphism rs7512998 is associated with systolic and diastolic blood pressure in 50-year-old subjects in the TAMRISK study. Between ages of 45- and 50 years blood pressure increased more in subjects with the C-allele. Age-related increase in blood pressure could have an inflammatory background where NLRP3 inflammasome action may be involved.

## Methods

### Subjects

TAMRISK study data was collected from periodic health examinations (PHE) done for 50-year-old men and women living in Tampere, a city in southern Finland with 220 000 inhabitants [16]. Basic evaluation in 1988–91 included an interview by a public health nurse. The interview was conducted using a structured questionnaire about health and health-related behavior. Current and previous diseases were identified based on self-report of diagnosis by a physician, including hypertension. Registration of blood pressure (mm of mercury) was made at the examination visit using a calibrated mercury sphygmomanometer using the first Korotkoff sounds for determination of systolic blood pressure and the last Korotkoff sounds for diastolic blood pressure. Subjects were seated in a chair with back support for at least 5 min prior to obtaining a measurement. Height (cm) and weight (kg) were recorded from which the body mass index (BMI) was calculated. Serum total cholesterol (mmoles/l) and glucose (mmoles/l) were measured after an overnight fast by standard techniques. Buccal swabs for DNA extraction and a permissions form to use PHE data were collected by mail separately of the physical examination. The DNA samples were collected during years 2006–2010. Informed consent was obtained from all participants. The Ethics Committees of the Tampere University Hospital and the City of Tampere approved the study.

Cases (*n* = 325) were subjects who had hypertension at the age of 50 years (as diagnosed by a physician) and for each case, at least one normotensive control subject (*n* = 444) with the same sex and similar smoking habits, was chosen in order of admission from a PHE cohort (*n* = 6000). The present study population at the age of 50 years thus included 769 subjects. Of these same individuals, we also analyzed the subpopulation of men and women who had available previous PHE data from the age of 45 years (*n* = 678), Both 50- and 45 year PHEs were conducted in a similar fashion by the same organization.

### Baseline measurements

#### Genotyping

DNA was extracted from buccal swabs using a commercial kit (Qiagen Inc., Valencia, Calif., USA). The samples were transferred into 96-well plates and genotyped for NLRP3 gene polymorphism rs7512998 at the KBioscience Institute (UK) using Competitive Allele Specific PCR (KASP) technique.

### Statistical analysis

Sample size was calculated by Quanto 1.2.4 (Copyright© 2000–2009, University of Southern California), with a choice of gene-only model. According to the World Health Organization a prevalence ratio of raised blood pressure in adults aged over 25 is about 40 % (http://www.who.int/gho/ncd/risk_factors/blood_pressure_prevalence_text/en/). The C-allele frequency of NLRP3 gene polymorphism rs7512998 was 0.1599 reported by National Center for Biotechnology Information (http://www.ncbi.nlm.nih.gov/snp/?term=rs7512998). Using 80 % power, a type I error rate of 0.05 and a two-sided statistical test, the required sample size per group was calculated to be 99. A total of 325 cases and 444 controls with successful genotyping were included in the final study population.

*T*-test for continuous variables and Chi-square test for categorical variables were applied for the comparison of groups. If the distribution was skewed, the analysis was performed using transformed values to approximately normalize the distribution. The analysis of variance for repeated measures was used to assess the differences in mean blood pressures between genotypes at the age of 45- and 50 years. Association analysis for NLRP3 gene rs7512998 genotype as a predictor of systolic and diastolic blood pressure was done using linear regression with gender, BMI and diagnosis of hypertension as cofactors. Analyses were carried out using SPSS 20.0 for Windows (SPSS Inc., Chicago, Illinois, USA).
